# The complete mitochondria genome of *Sipalus gigas* (Coleoptera: Curculionidae)

**DOI:** 10.1080/23802359.2020.1832591

**Published:** 2020-11-03

**Authors:** Jiayi Ma, Shaozhen Wang, Zuohua Huang, Xiaoqian Weng, Songqing Wu

**Affiliations:** a College of Forestry, Fujian Agriculture and Forestry University, Fuzhou, China; b Key Laboratory of Integrated Pest Management in Ecological Forests, Fujian Province University, Fujian Agriculture and Forestry University, Fuzhou, China

**Keywords:** *Sipalus gigas*, mitochondria genome, phylogenetic analysis

## Abstract

*Sipalus gigas* is the main pine-hole borer of Pinus. The length of the complete mitochondria genome of *S. gigas* was 17,120 bp with 33.6% GC content, there were 35 genes including 13 protein-coding genes (CDS), 20 transfer RNA genes (tRNAs), and two ribosomal RNA genes (rRNAs). This study provides useful genetic information for subsequent studying the prevention of *S. gigas*.

*Sipalus gigas* belongs to Curculionidae of Coleoptera, which is mainly distributed in Asian countries such as China, North Korea, and Japan (Furuta 1972). *Sipalus gigas* is one of the major pin-hole borers of Pinus, which including *Pinus massoniana*, *P. koraiensis*, and *P. tabulaeformis* (Yu et al. 2016). It has been seriously threatened the growth of pine trees and the processing of wood. Therefore, effective control of *S. gigas* is a urgent issue, while it is difficult to control *S. gigas* in production. Nowadays, biological control with bacteria toxins or fungi has been an important method to control insects. However, the genome of *S. gigas* have yet to be clearly determined. In order to provide useful genetic information for subsequent study of the prevention, the complete mitochondria genome of *S. gigas* was sequenced and assembled, as well as constructed phylogenies of *S. gigas* by Neighbor-Joining to understand the evolution relationship.

The *S. gigas* adults were collected from Lianjiang, Fujian Province, China (119° 38′25*″*E, 26°9′21*″*N) by the traps with sexual attractants. The specimens were stored in the Fujian Agriculture and Forestry University (SLX-202007).The total DNA was extracted from the legs of *S. gigas* by TruSeq DNA sample Preparation kit (Vanzyme, CHN) and purified by QIAquick Gel Extraction kit (Qiagen, GER). The mitochondrial genome was sequenced through Illumina Hiseq 2500 by Genesky Biotechnologies Inc. (Shanghai, China). In all, 566,589,34 clean reads were obtained through quality analysis and filtration. Then these clean reads were assembled by using *de novo* and the MITOS web server (Bernt et al. 2013; Hahn et al. 2013). And tRNA genes were predicted using tRNAscan (Lowe and Eddy 1997). The complete mitochondria genome length of *S. gigas* was 17,120 bp (GenBank accession no. MT809476). The GC content of the complete genome was 33.60% and there were 35 genes including 13 protein coding sequences, 20 tRNAs, and two rRNAs.

To further investigate the phylogenetic position of *S. gigas*, according to the genome sequence of *S. gigas*, the phylogenetic analysis was constructed with fourteen different species of Coleoptera by MEGA 6.0 (Tamura et al. 2013) using Neighbor-Joining tree model with 1000 bootstrap replicates. The Neighbor-Joining tree showed that *S. gigas* was closely related to *Sitophilus zeamais* and sister to *Myllocerinus aurolineatus* (Figure 1). The complete mitochondrial genome of *S. gigas* will provide useful genetic information to better understand the genetic evolution in *Sipalus*, as well as in *Curculionidae* and other insects of Coleoptera.

**Figure 1.**
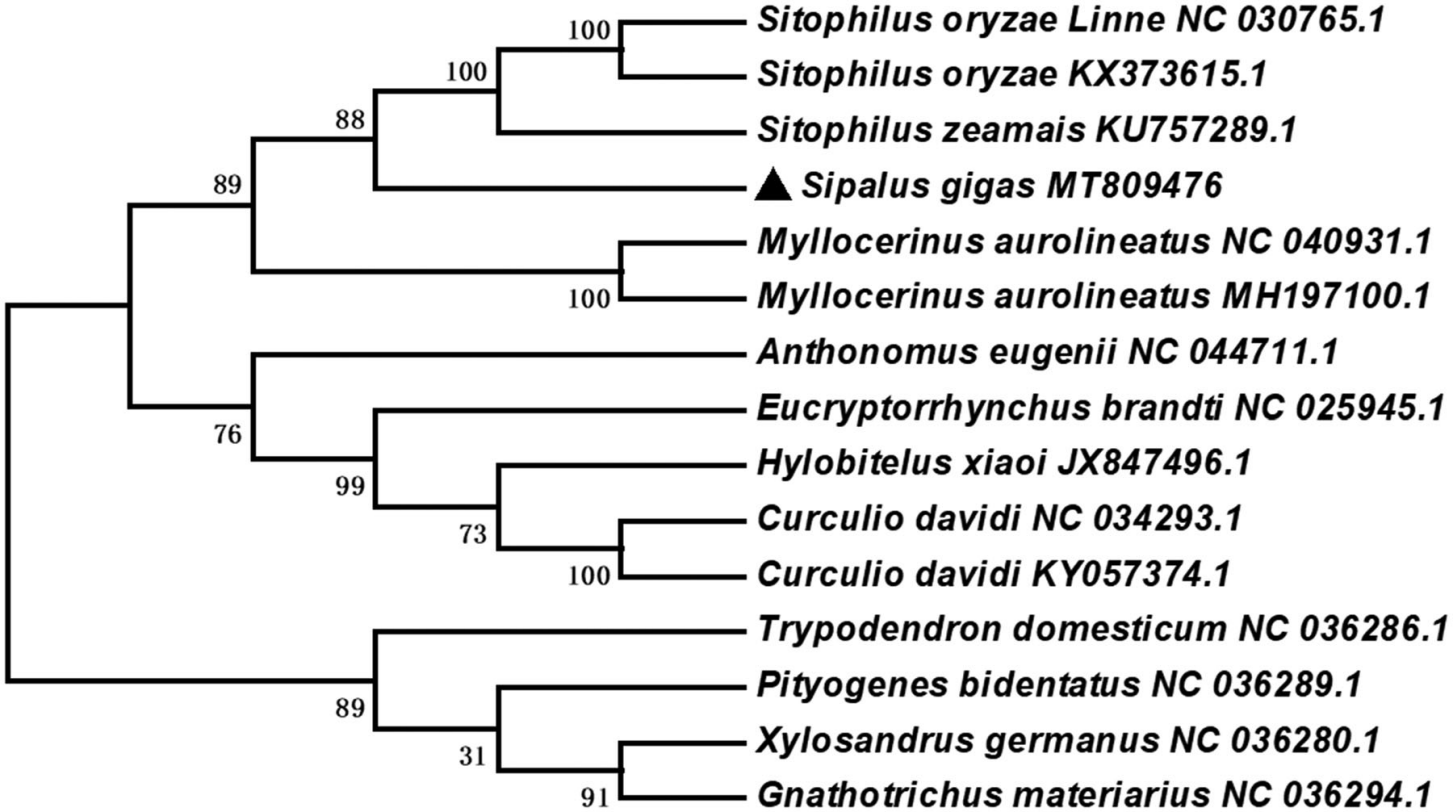
Neighbor-Joining tree of the *Sipalus gigas* and related 14 different species of Coleoptera based on the genome sequence. Numbers labeled on the branch are bootstrap values.

## Data Availability

The data that support the findings of this study are openly available in GenBank of NCBI at https://www.ncbi.nlm.nih.gov, reference number MT809476.
